# Optimizing Epicardial Restraint and Reinforcement Following Myocardial Infarction: Moving Towards Localized, Biomimetic, and Multitherapeutic Options

**DOI:** 10.3390/biomimetics4010007

**Published:** 2019-01-17

**Authors:** Claudia E. Varela, Yiling Fan, Ellen T. Roche

**Affiliations:** 1 Harvard-MIT Program in Health Sciences and Technology, Cambridge, MA 02139, USA; cvarela@mit.edu; 2 Institute for Medical Engineering and Science, Massachusetts Institute of Technology, Cambridge, MA 02139, USA; 3 Department of Mechanical Engineering, Massachusetts Institute of Technology, Cambridge, MA 02139, USA; yilingf@mit.edu

**Keywords:** ventricular restraint, infarct reinforcement, biomimetics

## Abstract

The mechanical reinforcement of the ventricular wall after a myocardial infarction has been shown to modulate and attenuate negative remodeling that can lead to heart failure. Strategies include wraps, meshes, cardiac patches, or fluid-filled bladders. Here, we review the literature describing these strategies in the two broad categories of global restraint and local reinforcement. We further subdivide the global restraint category into biventricular and univentricular support. We discuss efforts to optimize devices in each of these categories, particularly in the last five years. These include adding functionality, biomimicry, and adjustability. We also discuss computational models of these strategies, and how they can be used to predict the reduction of stresses in the heart muscle wall. We discuss the range of timing of intervention that has been reported. Finally, we give a perspective on how novel fabrication technologies, imaging techniques, and computational models could potentially enhance these therapeutic strategies.

## 1. Introduction and Rationale for Mechanical Reinforcement of the Left Ventricle Post-Myocardial Infarction

Over one million people suffer a myocardial infarction (MI), or heart attack in the U.S. every year [1]. An MI occurs when blood flow to a region of myocardium (heart muscle) is blocked, leading to cardiomyocyte death. The ischemic region, or infarct, loses its ability to contract, creating a mechanical disadvantage. Systolic function impairment can lead to the activation of compensatory mechanisms, pathological ventricular remodeling, and ultimately, heart failure (HF) [2].

For patients that survive an MI, preventing or hindering the development of HF is the main goal. Current clinical practices include medication and lifestyle changes in the post-MI stages. If patients progress to HF, left ventricular assistive devices (LVADs) can act as a bridge-to-transplant or a bridge-to-destination, but have inherent limitations, such as a propensity for thrombogenicity, and limited long-term success. Mechanical reinforcement strategies, where part or all of the heart is reinforced or restrained with an epicardially placed wrap or patch, have been proposed as promising therapeutic approaches. By supporting the diseased myocardium, these strategies can lead to an improved ventricular function by either preventing or hindering pathological left ventricle (LV) remodeling in the infarcted heart [3].

The epicardial restraint field was pioneered with cardiomyoplasty, a surgical procedure that aimed to restrain dilation and aid ejection in the failing heart by wrapping the epicardium with the latissimus dorsi muscle (from the posterior trunk) and stimulating the wrap to contract. Clinical studies showed that the efficacy of this procedure relied on the passive restraint exerted by the wrap [4]. Although the procedure was abandoned, it motivated further exploration into passive restraint. Initial devices were targeted at physically constraining the LV and treating end-stage HF. These efforts were later refocused at the earlier intervention of attenuating LV dilation post-MI [5].

One of the widely studied hypotheses in recent years has been whether the localized mechanical reinforcement of the infarct can limit LV dilation or prevent a progression to HF by limiting infarct expansion in the short-term following a heart attack [5]. With the contiguous development of the regenerative medicine field, several variations of local restraint arose and range from tissue-engineered or cell-seeded biomaterial patches to anisotropic reinforcement. For the purpose of this review, we limit our definition of local restraint to those devices, patches, or scaffolds—synthetic or naturally derived—that can exert a purely mechanical effect regardless of the presence of biological agents [5].

**Figure 1:**
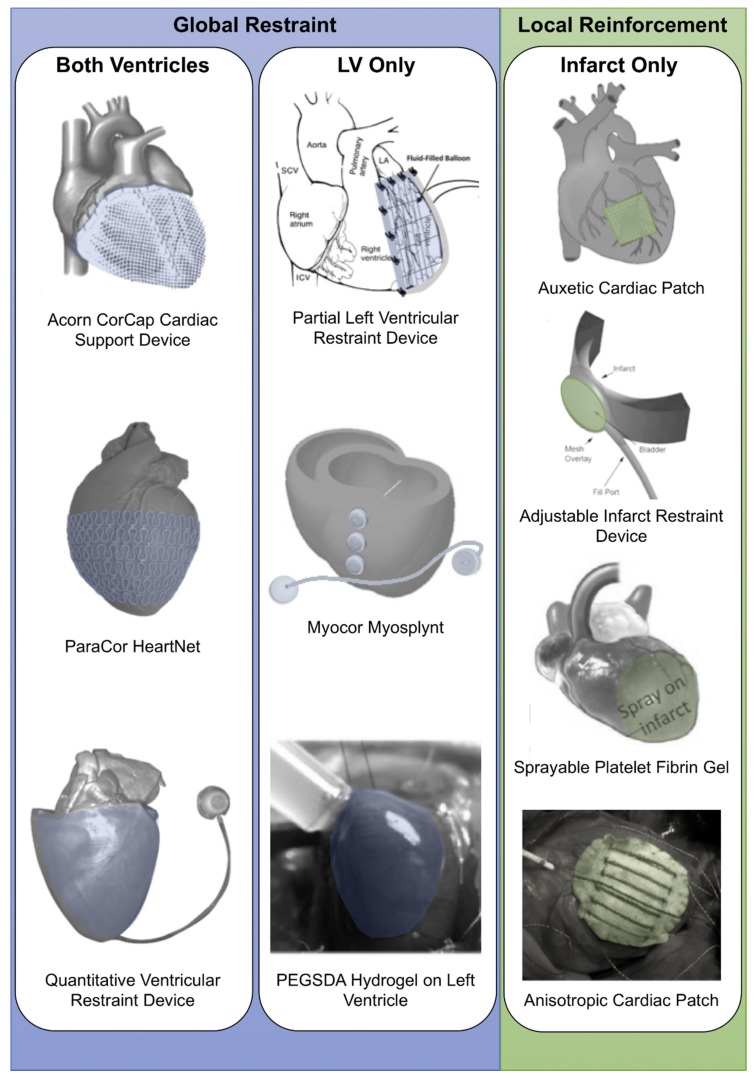
Visual representation of types of epicardial restraint and reinforcement devices including global restrain strategies (both ventricles and left ventricle (LV) only) and local reinforcement (infarct only). Images modified and reprinted from [8,11,12,13,14,15,16,17,18,19] with permission from Elsevier, Springer, Wolters Kluwer Health, Inc., and Mary Ann Liebert, Inc., respectively. PEGSDA: Poly(ethylene glycol) sebacate diacrylate.

Constant improvements of this therapeutic strategy are being reported in the literature. Motivated by one of the key limitations associated with early mechanical global and local restraint devices or patches—the inability to adapt the level of restraint—adjustable and quantifiable ventricular restraint [6] has recently been studied, and computational modeling has been used to determine the patch mechanical properties for optimal local reinforcement [7,8]. This review will discuss how mechanical support strategies post-MI have evolved in recent years. Epicardial implantable devices or patches, developed prior to 2013, have been thoroughly discussed in comprehensive reviews [3,5], and this review will focus on reviewing and discussing more recent studies on (i) the global epicardial restraint of one or both ventricles and (ii) the local reinforcement of the infarct region (Figure 1). We discuss how technological advancements in recent years have addressed previous limitations associated with post-MI cardiac restraint and reinforcement. Nonepicardial approaches, such as ventricular restoration procedures, perivascular devices and reinforcement via intramyocardial injection of biomaterials are beyond the scope of this review and for reviews in these topics we direct the reader to other work [8,9,10].

To identify studies to be included in this review—those describing epicardially placed mechanical support strategies post-MI—a chronological review of literature databases (i.e., Google Scholar, PubMed, and Web of Science), was conducted from 2013 to the present. The keywords used as search entries were ‘ventricular restraint’. Only search results pertaining to the cardiovascular field were considered and further filtered by criteria such as the nature of epicardial placement including global or local mechanical support.

## 2. Optimizing Biventricular Restraint Devices

Table 1 summarizes preclinical chronic results of biventricular restraint devices. The most extensively studied are the Acorn CorCap (Acorn Cardiovascular, Inc.) and the ParaCor HeartNet (Paracor Medical). The Acorn CorCap cardiac support device (CSD) is a mesh-like device made of knitted polyester that surrounds the heart [20]. The device is compliant and designed to provide greater diastolic support in the circumferential direction than in the apex-to-base direction. The CSD is first attached to the epicardium with a ring of sutures around the base of the heart, and then its two edges are sutured, creating a seam on the anterior wall. This allows for the adjustment of the CSD fit and allows a properly positioned device to bear enough of the end-diastolic load to reduce the degree of stretching and the magnitude of wall stress [20]. The CorCap’s efficacy to reduce or reverse the progression of heart failure was established with various ovine animal studies [21,22] and the subsequent demonstration of dilation prevention following an MI [23,24,25]. Promising results from preclinical testing led to a first-in-human safety study, where no device-related mortality was observed and improvements in the New York Heart Association (NYHA) classification and LV dilation were reported [26,27]. To follow, a multicenter randomized controlled trial initiated in 2004 and showed improvements in LV size and shape in patients with the CSD five years post-implantation. However, because the primary end-points of the trial were inconclusive (i.e., survival, changes in NYHA classification, or freedom from major cardiac procedures), the U.S. Food and Drug Administration (FDA) did not approve the device, primarily due to concerns of safety and efficacy [28,29].

The Paracor HeartNet CSD is a passive, biventricular restraint device consisting of a NiTinol wire mesh. It is designed to be flexible and elastic, to achieve a high degree of conformability to the heart when appropriately sized [13]. One benefit of the HeartNet is that the delivery system enables the implantation procedure to be less invasive than for other CSDs. Testing of the HeartNet in a canine HF model yielded encouraging results as hemodynamic parameters shifted back to pre-HF baseline over the course of the eight-week study. Prevention of dilation was observed in an ovine animal model of post-MI reinforcement six weeks after HeartNet implantation. Surprisingly, this did not translate to an improved cardiac function [34]. Initial safety and feasibility clinical testing of the HeartNet demonstrated low complication rates in 21 patients with NYHA II and III HF. This was followed by a 51-patient study which showed very promising improvements in cardiac function and no device morbidity. However, the major randomized, multi-institutional clinical trial with this device—Prospective Evaluation of Elastic Restraint to Lessen the Effects of Heart Failure (PEERLESS-HF)—was suspended because primary efficacy outcomes at six months were not met and all-cause mortality at 12 months post-device implantation was similar between the HeartNet and control groups [13].

### 2.1. Adjustability

One of the drawbacks of biventricular restraint is the possibility that the initial restraint level, while reinforcing the infarct region and preventing ventricular dilation, also inhibits diastolic filling. To address this, Ghanta et al. [12] developed a technique called quantitative ventricular restraint (QVR). This strategy is described by the implantation of a half ellipsoidal polyurethane balloon around both ventricles. The balloons are then inflated with saline to generate restraint, and the volume of saline in the reservoirs, and thus the level of restraint, can be modified through a subcutaneous port and indwelling catheter. By varying and optimizing the restraint level in an ovine animal model, they showed that improvements in cardiac output were achieved [35]. Later, the same group showed the effects of adjustable and measurable ventricular restraint (AMVR) on long-term LV remodeling [6], yielding the conclusion that the speed of reverse, positive remodeling increased with higher levels of restraint.

### 2.2. Adding Functionality

Following the validation of the aforementioned devices, the further optimization of ventricular support has been pursued. Efforts at enhancing this therapeutic strategy include the addition of functionality, the variation of the duration of restraint and the use of biomimetic materials to match the mechanical properties of heart muscle. For instance, in terms of added functionality, the validation of a new mechanism aiming to modulate diastolic filling was realized by Snowden et al. [36]. Further, Park et al. [37] developed an electric mesh that wraps around the heart to deliver electrical impulses to the entire ventricular myocardium. This wrap was constructed from silver nanowires embedded in a rubber polymer designed to conform to patient-specific three-dimensional (3D) anatomy. In a rat MI model, the group showed that the mesh exerts beneficial effects and integrates both structurally and electrically with the myocardium while preserving diastolic relaxation, reducing wall stress, and improving cardiac function.

Another knitted biventricular device was shown to positively impact LV remodeling and mitral regurgitation following MI creation. Okada et al. [33] report CSDs produced by a computer-assisted knitting machine (Shimaseiki Co., Wakayama, Japan) with two 5-0 polyester suture threads. After a three-month implantation in a canine MI model, they showed that LV remodeling was attenuated, while both LV and right ventricular (RV) systolic functions were improved in the CSD group compared to control. Diastolic function was not disturbed by the support device and mitral regurgitation was consistently prevented [33]. Another group later compared the effects of early and sustained restraint utilizing either a biodegradable or permanent textile CSD, respectively. They found that the biodegradable devices were more effective than the nonbiodegradable materials in improving systolic function while preserving diastolic function [32].

A biventricular CSD that simultaneously acted as a mechanical reinforcement device and a replenishable delivery system was first developed by Zhou [38], and termed active hydraulic ventricle supporting drug delivery system (ASD). As described by subsequent studies in the group, the ASD is a mesh-like device consisting of several hollow tubules which are interconnected but also have independent areas connected to external catheters. The hollow ASD tubes can be filled with various fluids to generate a pressure that will be transmitted to the epicardium. Additionally, the authors claim that a pneumatic pump can be attached externally with the ASD tubules and can deliver an adjustable and measurable optimized restraint at the beginning of therapy and as the heart shrinks during active reverse remodeling. Using the ASD, the group has claimed positive cardiac function effects in a rat MI model following repetitive delivery of *Salvia miltiorrhiza* [31] and bone marrow stem cells [30].

The use of soft robotics to manufacture implantable devices that can augment cardiac function was first introduced in 2016 by Roche et al. [39] and was continued by others [40,41]. Given the reported effects on initial mechanical reinforcement following MI, our group recently introduced a therapeutic paradigm in which a textile based soft robotic sleeve is implanted and allowed to biointegrate with the infarcted myocardium, providing mechanical reinforcement which will ideally limit LV dilation and downstream pathological remodeling. After the functional timeline for providing passive support has ended, the soft robotic sleeve would be coupled to the heart, then actuated to provide active augmentation of cardiac function [42].

## 3. Optimizing Left Ventricle Restraint Devices

Two clinically approved LV restraint devices are the Myosplint (Myocor) and the CardioClasp (Cardioclasp, Inc.). These devices interact with the LV only and their implantation procedure, which consists in reshaping the LV, does not allow them to be used as post-MI therapies, likely due to the risk of rupturing the weakened ischemic region [5]. However, their efficacy as treatments of HF has been demonstrated in computational models, animal studies, and clinical trials. The Myocor Myosplint is comprised of two rigid pads connected through the LV cavity. By increasing the tension between the two epicardial pads, the enlarged LV is reshaped into a bilobular ventricle with decreased chamber radius, which results in decreased wall stress [43]. Typically, three Myosplints are implanted in a longitudinal (apex-to-base) line on the LV, and the tension is adjusted until the radius of each lobe is approximately 80% of the radius of the dilated LV cavity. Animal testing in a canine model of rapid pacing heart failure yielded dramatic attenuation of chamber dilation four weeks post-implantation but no major functional improvements were observed between the treatment and control groups [11,44]. Initial clinical evaluation of the Coapsys, a similar device also developed by Myocor for mitral regurgitation, was promising and led to the initiation of Randomized Evaluation of Surgical Treatment for Off-Pump Repair of the Mitral Valve (RESTOR-MV) which ultimately was suspended due to financial limitations. However, analysis on two year follow-up data showed improved survival and decreased adverse events compared to standard surgical techniques [45,46].

The CardioClasp is another device that aims to reduce LV dilation by reshaping. It consists of two rigid bars connected through the LV cavity by an adjustable tether [47]. The bars are supposed to mimic the natural contour of the heart and are secured in place in a longitudinal (apex-to-base) line on the anterior and posterior LV walls. The tether is used to bring together the anterior and posterior bars until 30% reduction of the initial diastolic dimension is seen. Although multiple studies studying the acute effects of the CardioClasp in a canine HF model report a decrease in wall stress, increase in fractional area of contraction, increased systolic contractility, and positive chamber geometrical changes all relevant but no definite beneficial functional outcomes [47,48,49].

### 3.1. Adjustability

The group that developed the QVR and AMVR was the first to investigate whether ventricular restraint therapy was affecting both the RV and the LV in the same fashion and whether having the ability to adjust the level of restraint would positively change hemodynamic parameters [50]. In an acute study with healthy sheep, they found that the RV responds to restraint therapy differently than the LV by applying increasing levels of restraint and measuring hemodynamic parameters separately. They reported that increasing restraint level results in a nearly linear rise in RV filling pressure and at higher levels of restraint only RV filling was impaired. To correlate this with long-term outcomes, they simulated clinical restraint therapy in a sheep model of ischemic dilated cardiomyopathy with LV failure and showed that long-term restraint of the entire heart does not have any substantial impact on the RV [50]. In later years, they demonstrated that limiting restraint to the LV alone seems to be a superior therapeutic strategy given that it allows for a higher level of restraint before cardiac tamponade arises and for a potentially less invasive implantation procedure [14].

### 3.2. Adding Functionality

Consistent with design optimization strategies for biventricular restraint; adding functionality, varying the duration of restraint, and using biomimetic materials are features that have been explored. For instance, Kalogerakos et al. [44] developed a band of shape memory alloy fibers that would contract when electric current flows, similar to myocardium, and relax when the current flow is interrupted with the vision that this device could be anchored around the heart to assist in the pumping function, while simultaneously preventing LV dilation. By limiting LV restraint to the first week post-infarction with a biodegradable poly(ethylene glycol) sebacate diacrylate (PEGSDA) hydrogel, Vilaeti et al. [16] concluded that short-term LV restraint ameliorated LV remodeling at two weeks. Such effects were associated with a favorable inflammatory and foreign body response in the infarcted and noninfarcted zone. Interestingly, this study also evaluated a polyanhydroglucuronic acid/PEGSDA scaffold implanted immediately after MI that wrapped around the whole heart. The latter approach yielded better functional effects and less remodeling compared to the LV-only PEGSDA hydrogel alone and they hypothesize this is because increased fibroblast infiltration in the noninfarcted zone was observed earlier with the scaffold around the whole heart than with the hydrogel on the LV alone. However, the group acknowledges that this warrants further exploration and long-term evaluation [16] prior to translation to the clinic.

## 4. Computational Modeling

**Figure 2:**
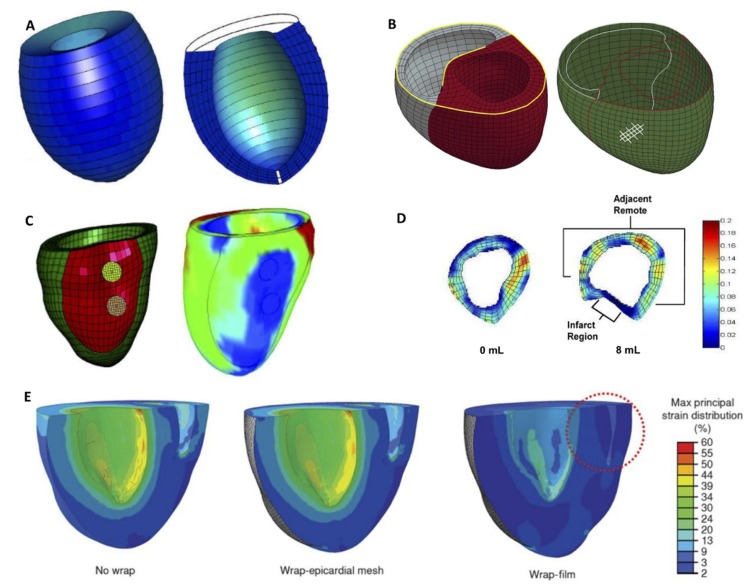
Finite element analysis (FEA)of ventricular support. (**A**) Three-dimensional FEA mesh of a dilated canine left ventricle (LV) in the unloaded state with a cross-sectional view of the interior cavity and wall (modified and reprinted from [51] with permission from Elsevier). (**B**) Biventricular FEA model with LV in red and right ventricle (RV) in grey (yellow lines are fully constrained). The green shell elements represent the Acorn cardiac support device (white lines on outside represent fiber orientations). Modified and reprinted from [52], with permission from Elsevier. (**C**) The Coapsys model showing the double pad side of the device. Infarct regions are colored with red while the remote regions are colored with green, myofiber stress at end systole in the virtual Coapsys model, showing a reduction of stress (in the blue area) compared to the control. Modified and reprinted from [55], with permission from Elsevier. (**D**) Device filling (8 mL) altered both the geometry and strain in the basal region where the device was positioned. Modified and reprinted from [18], with permission from Elsevier. (**E**) Maximum principal strain distribution without any wrap (left), with the epicardial mesh wrap (middle), and with the film wrap (right). Encircled area indicates RV collapse. Modified and reprinted from [37], with permission from the American Association for the Advancement of Science.

Finite element analysis (FEA) has been used to study various types of ventricular constraint devices. Figure 2 illustrates some of these studies and demonstrates the range of complexity of the models. Jhun et al. [51], created a simplified LV model to show that increasing passive epicardial restraint effectively eliminated end-diastolic myofiber stress while also reducing ejection fraction. The biventricular FEA model investigating the Acorn CorCap CSD also showed similar results [52]. Although it is outside the scope of this work, it is worth noting that FEA models of endocardial patch placement, and their effect on myofiber stress have also been described [53,54].

More sophisticated, patient-specific models were also used to study ventricular restraint devices. Carrick et al. [55] showed the mechanical benefit of the Coapsys. More recently, an FEA model created by Park et al. [37] examined the mechanical effect of cardiomyoplasty wraps on a rat heart. They showed that a full film wrap significantly reduced the ventricular compliance resulting in an elevated end diastolic pressure and decreased end diastolic volume while a mesh wrap only slightly altered the mechanical properties compared to the baseline. By incorporating fiber orientation derived from diffusion tensor imaging (DTI), Sack et al. [56], used a four-chamber human heart model to demonstrate the benefits of a left ventricular assist device on myocardial wall stress. To the best of our knowledge, no literature simulates local reinforcement therapeutic modalities discussed in the subsequent section utilizing FEA models.

## 5. Optimizing Local Reinforcement: Multifunctional, Biomimetic and Adjustable Devices

### 5.1. Overview and Timing of Intervention

**Figure 3:**
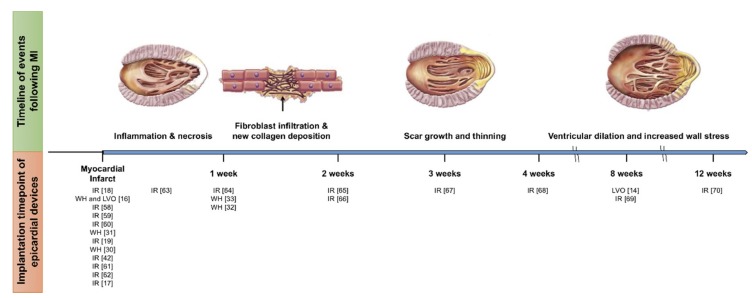
Side-by-side visualization of gross post-myocardial infarction (MI) left ventricular (LV) remodeling and intervention timing of epicardial devices/patches implantation. Illustration modified and reprinted from [71], with permission from Elsevier. IR: Infarct reinforcement; LVO: LV only restraint; WH: Whole heart restraint.

Locally restraining the infarct region to prevent infarct expansion and post-MI remodeling using synthetic patches has been evaluated as early as 1999 [57]. In recent years, an abundance of studies report on the effect on cardiac function following implantation of cardiac patches, naturally derived or synthetic scaffolds, and devices mechanically reinforcing the infarct region. Chronic studies are summarized in Table 2. Some of these were originally intended to provide localized mechanical reinforcement; others were implanted primarily for the delivery of bioagents to the infarct region but their attachment to the epicardium simultaneously provides a mechanical effect. Because local infarct restraint intends to prevent infarct expansion most of which occurs early post-MI, the majority of scaffolds, patches and devices evaluated have been implanted immediately post-MI [17,18,19,42,58,59,60,61,62]. However, some groups have seen benefits in cardiac function upon patch implantation at latter timepoints post-MI [63,64,65,66,67,68,69,70] ranging from four days to 12 weeks (Figure 3).

### 5.2. Adding Functionality

Due to increasing interest in the promise of cardiac regeneration over the last few decades, a multitude of studies have utilized tissue engineering patches or biological or synthetic matrices loaded with therapeutic agents while providing local mechanical reinforcement to the post-MI ventricle. For instance, Rodness et al. [63] observed that the implantation of a calcium–alginate microsphere patch, with and without vascular endothelial growth factor (VEGF) and restrained by a chitosan sheet four days following MI, yielded better cardiac function as compared to rats with only a chitosan sheet. This group also reported on the added benefit that including the delivery of bioagents can have in the revascularization of ischemic myocardium in addition to mechanical reinforcement. They observed that VEGF-loaded microsphere patches had a thicker scar with higher capillary density in the border zone as compared to rats without VEGF in the microsphere patch.

One group has conducted studies utilizing a commercially available extracellular matrix (ECM) biomaterial patch procured from porcine small intestine submucosa, named CorMatrix–ECM (CorMatrix Cardiovascular, Inc.) for its intrinsic bioactive properties and as a carrier of bioagents. For instance, in one study, they enhance the CorMatrix–ECM patch with basic fibroblast growth factor (bFGF), a potent inhibitor of fibrosis, to study its effects on myocardial fibrosis, chamber dilation, and progression to heart failure in a rat MI model 16 weeks post-treatment. They report improved ejection fraction following treatment bFGF-enhanced CorMatrix–ECM patch when compared to the control group as well as a significant reduction in LV dilation when compared to both the control group and the nonenhanced CorMatrix–ECM patch, suggesting the addition of bFGF was beneficial [67]. On a different study, the group used an ischemia reperfusion porcine model to show that the application of a biologic ECM construct, such as the CorMatrix-ECM, significantly increases endogenous myocardial recovery and vasculogenesis [60]. They confirmed that bioactive properties within the acellular ECM biomaterial are essential for functional benefits and positive impacts on remodeling to be observed using a rat MI model [73]. Although the biological effect ECM patches impart on the damaged myocardium are discussed, further understanding of the mechanical impact these bioactive scaffolds provide could lead to the further optimization of this combined therapeutic strategy. Indeed, one study by D’Amore et al. [66], evaluated the incorporation of ECM bioactivity and specific mechanical patch anisotropy in a rat MI-model. They showed that the implantation of a bilayered scaffold with isotropic mechanics and a cardiac ECM-enriched layer mitigates adverse remodeling 10 weeks post-MI by decreasing LV global mechanical compliance, mitigating scar formation and LV wall thinning, and promoting angiogenesis.

In addition to delivering therapeutic proteins, epicardially placed synthetic and natural patches can be used as scaffolds to deliver cells, or act as scaffolds for transplanted cells, enabling delivery of paracrine factors to the infarcted heart. In one such study, researchers implanted an elastic, biodegradable cardiac patch made of poly(ε-caprolactone)/gelatin (PG) nanofibers and loaded with mesenchymal stem cells (MSCs) one week post-MI in rats [64]. The combination of the PG patch and MSCs effectively restricted expansion of the LV wall, reduced scar size, and increased the density of vessels at the infarct site after four weeks of implantation. Interestingly, they attribute the mechanical effects of limiting LV expansion to the PG nanofibrous patches and the cardiomyogenic and angiogenic effects they observe to the MSCs loaded in the patch.

In order to address some of the limitations associated with the sustained delivery of biological agents and cell therapy to the epicardial surface, our group developed a replenishable reservoir that, when implanted into the epicardial surface following MI, improved cardiac function following the repeated administration of MSCs [61]. We observed that the implantation of the device itself without the delivery of MSCs also had a beneficial effect in cardiac function, likely because the materials used to construct the reservoir provided mechanical reinforcement at the infarct zone upon MI creation.

Apart from the incorporation of biological therapy to cardiac patches, two different groups also utilized conductive nanomaterials to improve electrical implant integration and compatibility with the myocardium as well as cardiac function [17,65]. One of them, used single-walled carbon nanotubes (SWNTs) incorporated into gelatin hydrogel scaffolds for the construction of engineered cardiac tissues. They found that their tissues structurally integrate with the host myocardium achieving a bidirectional migration of cells. Auxetic cardiac patches have also been developed, with tunable mechanical and conductive properties aiming to provide both mechanical reinforcement and allow electrical conduction to prevent arrhythmias [17].

### 5.3. Adjustability

As with global restraint devices, an approach taken to optimize the local reinforcement of the infarct was to make the level of restraint adjustable to better understand how this parameter affects LV function and remodeling. In 2013, Koomalsingh et al. [18] used a device designed to produce the variable alteration of infarct stiffness and geometry made of a polypropelene mesh and inflatable balloon catheter. In a pig MI model, they showed that an optimized level of infarct restraint can limit adverse LV remodeling after MI. In 2018, Kataoka et al. [69] validated another adjustable device made of a polymer filled mesh with the objective to reverse LV remodeling and reduce ischemic mitral regurgitation (IMR) in a sheep model. At implantation eight weeks into IMR, the adjustment of the device was done such that IMR was minimized and in a chronic study the group showed that this initial mechanical reinforcement led to a reduction of end-systolic and diastolic LV volumes. Another group pursued a study to test if the minimally invasive delivery of an inflatable localized device to treat ICM was feasible [70]. Their device was made of a heavy-duty 2.5 × 2.5 cm neoprene rubber inflatable bladder that was positioned centrally within the infarct and then secured to the surrounding border zone myocardium with polypropylene mesh. They validated that a minithoracotomy insertion of such device in a pig model was possible and by providing active assistance to the infarct region showed dramatic improvements in systolic function using magnetic resonance imaging [70].

The aforementioned studies use patches, scaffolds, and devices localized to the infarct area to better limit LV remodeling with mechanical reinforcement in conjunction with biological therapy delivery or electrical coupling, and in some cases, with adjustable mechanical restraint. Purely biomechanical studies have also been conducted to isolate and elucidate how global and local heart mechanics alter scar composition, a key determinant of the degree of pathological remodeling. Recently, Caggiano et al. [62] tested how modulating the local mechanical environment of the infarct altered scar collagen turnover, accumulation, and alignment in a rat MI model. Using anisotropic Dacron patches that eliminated circumferential strain in the infarct, the group showed that scar collagen aligned parallel to the regional strain experienced. This preferential mechanical reinforcement did not reduce the collagen area fraction but lead to a significantly reduced LV wall thickness when compared to untreated controls. These findings call for the consideration of regulating regional mechanics at the infarct zone when optimizing infarct reinforcement approaches.

## 6. Conclusions and Future Perspectives

As described in this review, ventricular restraint and infarct reinforcement are promising therapeutic approaches that can reduce or prevent LV remodeling post-MI. Global, biventricular restraint was the first approach tested clinically and has been studied in detail. Adjustability incorporated in these devices is highly beneficial, as the optimal level of restraint required to provide mechanical reinforcement yet avoid restriction of filling and tamponade can change over time as the heart adapts post-therapy. Currently, devices that can quantify and adjust the level of restraint consist of simple, fluid-filled bladders. Potentially, with the advent of 3D printing and soft robotic techniques, we can achieve a higher fidelity biomimetic mechanical reinforcement that conforms to the heart, moves with the heart wall, and has adaptable mechanical properties that can be tuned and adjusted over time. Using soft robotics, we could also achieve a finer control over spatial regions of supporting structures, for example to provide variable stiffness or anisotropy to optimize regional mechanical benefit to the infarcted heart. These techniques increase the potential design space for smart, customized biventricular reinforcement. Of course, further study is warranted to see the level of improvement in functions enabled by this higher granularity, and how much clinical improvement would result. In terms of univentricular support, various studies corroborate the fact that the ventricles respond differently to restraint. This further motivates the design of a smart, tunable device that that imparts different levels of restraint to each ventricle.

In terms of local post-MI reinforcement, literature in recent years has largely focused on the implantation of cardiac patches or scaffolds that also exert a mechanical effect. While results are promising, tools for minimally invasive delivery are desirable to accelerate these strategies along the path to clinical translation. The use of anisotropic patches that control how the infarct area is mechanically deformed demonstrate the importance of the effect of the mechanical environment on scar structure and LV remodeling. To date, researchers have limited deformation in circumferential or apicobasal directions. Finer control over material anisotropy in reinforcing patches may enable studies with patches with anisotropic properties parallel or perpendicular to the epicardial fibers. Indeed, further study is necessary to fully understand how patient-specific reinforcement might yield optimal therapeutic effects. Incorporating high resolution imaging techniques and computational modeling to make patient-specific patches could help elucidate whether this level of customization leads to better functional effects post-MI or if a more global approach to mechanical intervention is sufficient to see functional benefits. For example, patches that include specific fiber orientation could be designed based on DTI data, and computational modeling, as described in this review, could aid with optimization of patch/device design. Indeed, in vivo tracking of structural changes in the infarct region and the surrounding tissue has been reported in various studies using DTI [74,75,76,77]. Combining temporally dependent fiber orientation data with FEA simulation could aid in the design, testing, and optimization of local and global reinforcement strategies. Finally, a polytherapeutic approach is likely the most beneficial for scar modulation post-MI. As described here, many studies have included biological or electrical functionality in addition to mechanical reinforcement and while results combining therapeutic strategies are promising, further study of clinically relevant scenarios where the impact of added patch/device functionality and adjustability can be quantified in terms of overall therapeutic effect are needed. Further study of the temporal sequence of these strategies, and their interplay, should be a focus of future work since it is likely that optimizing a combination of approaches will yield the highest clinical benefit.

## Figures and Tables

**Table 1:** Summary of chronic epicardial global restraint studies (those reviewed in [5] are not included).

Type of Restraint	Animal	Infarct/HF Model	Follow-Up Time Post-MI	Restraint Device or Patch Material	EDV	ESV	EF	SV	CO	FS/FAS/WT	d*P*/d*t*	ESPVR	Reference
Global (both ventricles)	Male Sprague Dawley rats	LAD ligation	30 days	ASD device (biocompatible silicone) + BMSC	-	-	-	-	-	-	↑	-	[30]
Global (both ventricles)	Male Sprague Dawley rats	LAD ligation	30 days	ASD device (silicone) + *Salvia miltiorrhiza*	-	-	-		-	-	↑	-	[31]
Global (both ventricles)	Beagle dogs	LAD diagonal ligation	12 weeks	Biodegradable polyglycolic acid suture knitted support device	↔	↔, ↓ ΔLVESV	↑ LVEF		-	-	↔	-	[32]
				Nonbiodegradable polyethylene terephthalate suture knitted support device	↔, ↓ ΔLVEDV	↔, ↓ ΔLVESV	↔ LVEF		-	-	↔	-	
Global (both ventricles)	Beagle dogs	Posterior wall infarction by ligation of proximal/distal branches of left diagonal, obtuse marginal and posterior coronary arteries	3 months	CSD knitted dog mesh (polyester sutures)	↔	↔	↑		-	-	-	*E*_max_ ↑	[33]
Global (both ventricles)	Wistar rats	LAD ligation	15 days	PEGSDA-coated polyanhydroglucuronic-acid scaffold	-	-	↑		-	-	-	-	[16]
Global (LV only)	Wistar rats	LAD ligation		PEGSDA hydrogel	-	-	↑		-	-	-	-	

ASD: Active hydraulic ventricle supporting drug delivery system; BMSC: Bone marrow derived stem cells; CO: Cardiac output; CSD: Cardiac support device; d*P*/d*t*: Rate of systolic pressure generation; *E*_max_: End-systolic elastance; EDV: End-diastolic volume; EF: Ejection fraction; ESPVR: Slope of the end-systolic pressure–volume relationship; ESV: End-systolic volume; FAS: Fractional area shortening; FS: Fractional shortening; HF: Hart failure; LAD: Left anterior descending coronary artery; LV: Left ventricle; LVEF: Left ventricular ejection fraction; LVESV: Left ventricular end-systolic volume; MI: Myocardial infarction; PEGSDA: Poly(ethylene glycol) sebacate diacrylate; SV: Stroke volume; WT: Wall thickening. ↑: Increased; ↔: No change; ↓: Decreased.

**Table 2:** Summary of chronic epicardial local reinforcement studies (those reviewed in [5] are not included).

Animal	Infarct/HF Model	Follow-Up Time Post-MI	Restraint Device or Patch Material	EDV	ESV	EF	SV	CO	FS/FAS/WT	d*P*/d*t*	ESPVR	Reference
Male C57BL mice	LAD ligation	14 days	Medical polyester mesh or silicone patch									[42]
Female Sprague Dawley rats	LAD ligation	28 days	Therepi (TPU and polycarbonate membrane) + rMSC paracrine factors			↑			↑ FS			[61]
Sheep	LCx ligation/ischemic mitral regurgitation	16 weeks	Poly-mesh (polyester mesh containing polyacrylamide granules with outer border of polyester fabric)	↓	↓	↔ LVEF				↔	↑ *E*_max_	[69]
Male Sprague Dawley rats	LAD ligation	6 weeks	PDMS-coated Dacron patch						↓ infarct WT			[62]
Male Sprague Dawley rats	LAD ligation	2 weeks	Polyaniline and phytic acid grown on micropatterned chitosan films	↔	↔				↔ FS			[72]
Male New Zealand white rabbits	Left posterolateral/lateral coronary artery ligation	6 weeks	PLLA patch + GCSF	↓	↓	↑	↑	↑	↑ FS			[68]
Male CD1 mice	LAD ligation	21 days	Platelet fibrin patch			↑			↑ FS			[19]
Rat	LAD ligation	14 weeks	Inactivated SIS-ECM patch	↓		↑			↑ WT	(d*P*/d*T*)/LVEDV ↑	↑	[73]
Female Lewis rats	Proximal Left Coronary Artery ligation	10 weeks	Biodegradable PECUU + isotropic ECM enriched layer						↑ FAC			[66]
Female Sprague Dawley rats	LAD ligation	1 month	Ca–alginate microsphere patch covered in chitosan sheet						↑ FS			[63]
Male Landrace pigs	Ischemia reperfusion	6 weeks	SIS-ECM patch	↔	↔	↔						[60]
Male Mongrel dogs	LAD ligation	8 weeks	Longitudinally inextensible knitted polyester and bovine collagen patch	↔	↔				↔ infarct WT			[59]
Male Fischer CDF rats	LAD ligation	16 weeks	bFGF-enhanced SIS-ECM patch	↓		↑					↔ ESPVR and EDPVR	[67]
Male Sprague Dawley rats	LAD ligation	6 weeks	SWNT/gelatin hydrogel patch + neonatal rat cardiomyocytes			↑			FS ↑	↑		[65]
			Gelatin hydrogel patch + neonatal rat cardiomyocytes			↑			FS ↑			
			Gelatin hydrogel patch + neonatal rat cardiac fibroblasts			↑			FS ↑			
Female Sprague Dawley rats	LAD ligation	5 weeks	Biodegradable PG nanofibrous patch + rMSC	↓	↓	↑			FS ↑			[64]
Pigs	LCx ligation	4 weeks	Polypropylene mesh covering balloon catheter	↑	↑	↑						[18]
Female Wistar rats	Cryo-injury of LV	8 weeks	Chitosan–HYA/SF patches						↑ WT, ↑ FS			[58]

bFGF: Basic fibroblast growth factor; CO: Cardiac output; d*P*/d*t*: Rate of systolic pressure generation; *E*_max_: End-systolic elastance; EDPVR: Slope of the end-diastolic pressure–volume relationship; EDV: End-diastolic volume; EF: Ejection fraction; ESPVR: Slope of the end-systolic pressure–volume relationship; ESV: End-systolic volume; FAS: Fractional area shortening; FS: Fractional shortening; GCSF: Granulocyte colony-stimulating factor; HYA: Hyaluronan; LAD: Left anterior descending coronary artery; LCx: Left circumflex artery; LV: Left ventricle; LVEF: Left ventricular ejection fraction; MI: Myocardial infarction; PECUU: Poly(ester carbonate urethane)urea; PDMS: Polydimethylsiloxane; PG: Poly(ε-caprolactone)/gelatin; PLLA: Poly-l-lactide; rMSC: Rat mesenchymal stem cells; SF: Silk fibroin; SIS-ECM: Small intestinal submucosal extracellular matrix; SV: Stroke volume; SWNT: Single-walled carbon nanotubes; TPU: Thermoplastic polyurethane; WT: Wall thickening. ↑: Increased; ↔: No change; ↓: Decreased.
